# Analysis of Chromosomal Copy Number in First-Trimester Pregnancy Loss Using Next-Generation Sequencing

**DOI:** 10.3389/fgene.2020.545856

**Published:** 2020-10-20

**Authors:** Lei Fan, Jianli Wu, Yuanyuan Wu, Xinwei Shi, Xing Xin, Shufang Li, Wanjiang Zeng, Dongrui Deng, Ling Feng, Suhua Chen, Juan Xiao

**Affiliations:** Department of Obstetrics and Gynecology, Tongji Hospital, Tongji Medical College, Huazhong University of Science and Technology, Wuhan, China

**Keywords:** first-trimester pregnancy loss, chorionic villi, next generation sequencing, fetal chromosomal abnormality, copy number variation

## Abstract

Embryonic chromosomal abnormality is one of the significant causative factors of early pregnancy loss. Our goal was to evaluate the clinical utility of next-generation sequencing (NGS) technology in identifying chromosomal anomalies associated with first-trimester pregnancy loss. In addition, we attempted to provide fertility guidance to couples anticipating a successful pregnancy. A total of 1,010 miscarriage specimens were collected between March 2016 and January 2019 from women who suffered first-trimester pregnancy loss. Total DNA was isolated from products of conception, and NGS analysis was carried out. We detected a total of 634 cases of chromosomal variants. Among the 634 cases, 462 (72.9%) displayed numerical variants including 383 (60.4%) aneuploidies, 44 (6.9%) polyploidies, and 34 (5.5%) mosaicisms. The other 172 (27.1%) cases showed structural variants including 19 (3.0%) benign copy number variations (CNVs), 52 (8.2%) pathogenic CNVs, and 101 (16%) variants of unknown significance (VOUS) CNVs. When maternal age was ≥ 35 years, the sporadic abortion (SA) group showed an increased frequency of chromosomal variants in comparison with the recurrent miscarriage (RM) group (90/121 vs. 64/104). It was evident that the groups with advanced maternal age had a sharply increased frequency of aneuploidy, whatever the frequency of pregnancy loss (71/121 vs. 155/432, 49/104 vs. 108/349). Our data suggest that NGS could be used for the successful detection of genetic anomalies in pregnancy loss. We recommend that fetal chromosome analysis be offered routinely for all pregnancy losses, regardless of their frequency.

## Introduction

Pregnancy loss occurs in nearly 10–15% of all clinically confirmed pregnancies, primarily during the first trimester (Hertz-Picciotto and Samuels, 1988; Rai and Regan, 2006). Approximately 50–60% of all early pregnancy losses may be attributed to fetal chromosomal abnormalities (Goddijn and Leschot, 2000; van den Berg et al., 2012). While most abnormalities include numerical variants such as trisomy, polyploidy, and monosomy X, structural anomalies constitute a small portion of these aberrations (Kalousek et al., 1993; Stephenson et al., 2002).

Over the years, researchers have tried to elucidate the genetic causes of pregnancy loss. G-band karyotyping is a traditional cytogenetic technique employed for the genetic analysis of abortus (Israel et al., 1996; Carp et al., 2001). However, G-band karyotype analysis cannot detect chromosomal aberrations of less than 5 Mb (Saldarriaga et al., 2015). Moreover, failure of culture, contamination from cells of maternal origin, and suboptimal chromosome preparation may lead to erroneous results (Robberecht et al., 2009).

Copy number variation (CNV) is the most common form of structural variation and refers to the duplication or deletion of DNA segments greater than 1 Kb. CNVs include insertions, deletions, and duplications of genomic regions (Redon et al., 2006). In human populations, CNVs are present at a high frequency (10%) (Iafrate et al., 2004). Although pathogenic CNVs account for about 1% of individuals, specific CNVs are associated with diseases such as cancer, autism, Alzheimer’s disease, and neuropsychiatric disorders (Erikson et al., 2015). Based on these reports, there is an increased interest in elucidating whether a correlation exists between abnormal CNVs and early pregnancy loss. Chromosomal microarray analysis (CMA), including array comparative genomic hybridization (a-CGH) and single-nucleotide polymorphism (SNP) microarray, is widely employed in the detection of CNVs (Bell et al., 2001; Rosenfeld et al., 2015). Unfortunately, array-based techniques are unable to identify shorter CNVs. Moreover, neither a-CGH analysis nor SNP microarray can identify balanced translocations, inversions, or tetraploidies (McQueen and Lathi, 2019). Next-generation sequencing (NGS) is a breakthrough technology that has revolutionized clinical and basic science research (Schuster, 2008). Unlike Sanger sequencing, NGS technology has a higher resolution (<10 kbp), making it possible to detect smaller CNVs (<10 kbp) (Korbel et al., 2007). Moreover, NGS can identify more mosaicisms (Sahoo et al., 2017).

In reproductive medicine, NGS is increasingly being used for procedures such as pre-implantation genetic screening (PGS) and the detection of chromosomal abnormalities in blastocysts derived from embryos fertilized *in vitro* (Friedenthal et al., 2018; Palmerola et al., 2019). Given the usefulness of NGS in detecting chromosomal abnormalities in embryos, it is expected to improve the prediction of pregnancy outcomes (Friedenthal et al., 2018). In several studies, chromosomal analysis of the products of conception was performed using NGS (Liu et al., 2015; Qiao et al., 2016; Shen et al., 2016). However, most of these studies did not have sufficient enough sample sizes to draw conclusive inferences.

In the present study, we used NGS to identify CNVs in the chorionic villus samples from women who had undergone early spontaneous pregnancy loss. Through this study, we evaluated the feasibility of NGS in the detection of chromosomal abnormalities in first-trimester pregnancy loss and elucidated the associations, if any, between the chromosomal abnormalities and the pregnancy loss.

## Materials and Methods

### Subject Enrollment and Sample Collection

The study was conducted at the Department of Obstetrics & Gynecology, Tongji Hospital, Tongji Medical College, Huazhong University of Science and Technology between March 2016 and January 2019. Women who suffered from spontaneous pregnancy loss before 14 weeks of gestational age and consented to participate in the study to determine the possible genetic anomalies were enrolled. When spontaneous pregnancy loss occurs at least twice, it is classified as recurrent pregnancy loss [recurrent miscarriage (RM)]. All the patients signed informed consent forms. The study was approved by the medical ethics committee of Tongji Hospital, Tongji Medical College, Huazhong University of Science and Technology.

### Sample Preparation for Next-Generation Sequencing

A total of 1,010 fresh chorionic villi were obtained by the clinical routine uterine apoxesis, carefully separated from maternal decidua. Genomic DNA was extracted from the villi using the DNeasy Blood and Tissue Kit (Qiagen GmbH, Hilden, Germany). The DNA was quantified using the NanoDrop spectrophotometer (Thermo Fisher Scientific, Wilmington, DE, United States), and the quality was assessed by agarose gel electrophoresis. All DNA samples that passed the quality control measures (concentration > 50 ng/μl; OD260/OD280 > 1.8; OD260/OD230 > 1.5) were amplified by quantitative polymerase chain reaction (qPCR). Finally, samples were sent to Beijing Berry Genomics Co., Ltd., for CNV sequencing using the NextSeq CN500 platform (Illumina Inc.).

### Chromosomal Copy Number Sequencing by Next-Generation Sequencing

NGS was performed according to the normative protocol for specific procedures (Liang et al., 2014; Dong et al., 2016). The annotation and interpretation were carried out based on the guidelines of the American College of Medical Genetics and Genomics (Kearney et al., 2011). A CNV is defined as a segment of DNA of at least 1 kb that differs in copy number when compared to a representative reference genome. The CNVs were classified into benign, pathogenic, and variants of uncertain significance (VOUS) types as per American College of Medical Genetics and Genomics (ACMG) standards and guidelines (Riggs et al., 2020). All the original data were deposited in our repository.

### Statistical Analyses

We used IBM SPSS Statistics 24 software for statistical analysis. When the sample size is large enough, the chi-square (χ^2^) test was used to compare the rates between the two groups. If any prediction frequency is less than 5, we analyzed the results of the Fisher’s exact test. Results with *P* < 0.05 were considered statistically significant.

## Results

### Characterization of Chromosomal Results

**FIGURE 1:**
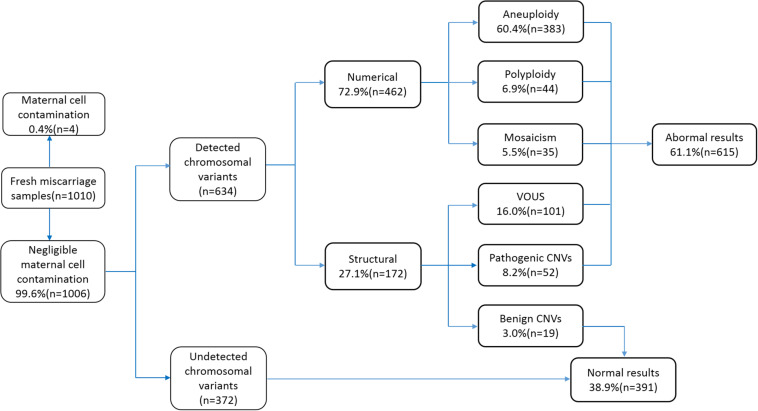
Flowchart depicting the details of samples analyzed in this study and summary of the characterized chromosomal copy number variations (CNVs). VOUS, variants of unknown significance.

We collected 1,010 samples, of which four (0.4%) samples were excluded from the study due to contamination by maternal cells. The remaining 1,006 (99.6%) cases were subjected to CNV analysis by NGS. The sample characteristics and results are summarized in Figure 1. The gestational age ranged between 6 and 14 weeks (average 9 ± 1 weeks). Among the 1,006 cases, 372 cases did not show any chromosomal variants, while the other 634 cases presented various chromosomal variants. The detected variants were categorized as numerical variants (462/634, 72.9%) and structural variants (172/634, 27.1%). Furthermore, numerical variants included 383 aneuploidy (60.4%), 44 polyploidy (6.9%), and 35 mosaicisms (5.5%). Structural variants were divided into two groups: deletions/duplications (CNVs ≥ 10 Mb) and microdeletion/microduplication (CNVs < 10 Mb), including 19 benign CNVs (3.0%), 52 pathogenic CNVs (8.2%), and 101 VOUS CNVs (16%). Notably, benign CNVs were considered to be normal chromosomal variants.

### Characterization of Chromosomal Anomalies

**FIGURE 2:**
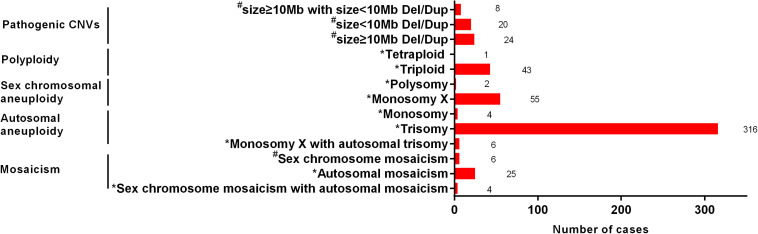
The type and number of cases of chromosomal anomalies. *, The possible cause of pregnancy loss; ^#^, not the possible cause of pregnency loss.

The type and number of cases of chromosomal anomalies are summarized in detail in Figure 2. The most common numerical abnormality was aneuploidy, divided into autosomal aneuploidy (four monosomies and 316 trisomies), sex chromosomal aneuploidy (two polysomies and 55 monosomy X), and six cases with both monosomy X and autosomal trisomy. The second most common type of numerical abnormality was polyploidy, including 43 triploidy and one tetraploidy. Mosaicism was shown in 25 autosomal mosaicisms, six sex chromosomal mosaicisms, and four mixed mosaicisms. Pathogenic CNVs were detected in 52 cases, including 24 cases with CNVs ≥ 10 Mb, 20 cases with CNVs < 10 Mb, and eight cases with both mentioned above.

### Frequency of Chromosomal Aneuploidy According to the Types of Pregnancy Loss and Maternal Age

**FIGURE 3:**
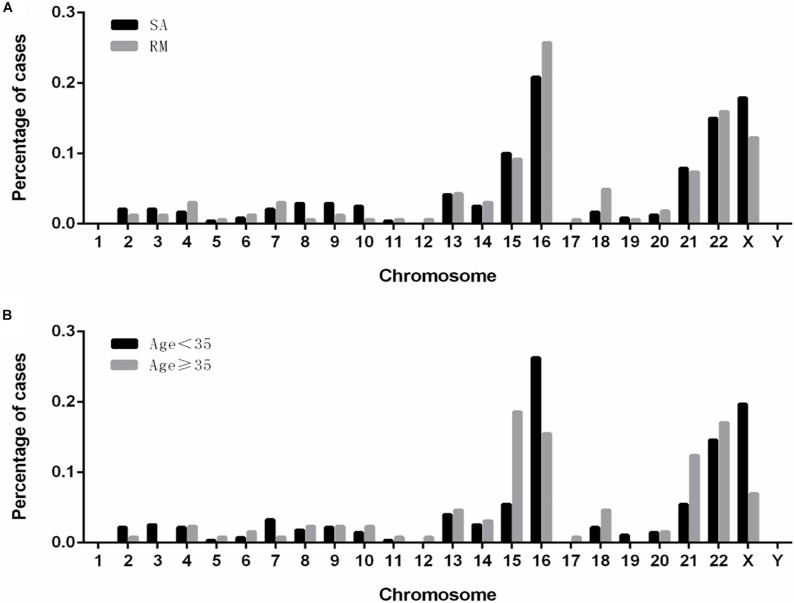
The distribution and frequencies of chromosomal aneuploidies. **(A)** Frequency of aneuploidies in patients with spontaneous and recurrent miscarriage. **(B)** Frequency of aneuploidy in patients of younger (<35 years) and advanced (≥35 years) maternal age. SA, sporadic abortion; RM, recurrent miscarriage.

We analyzed the distributions of aneuploidies observed in our cases (Figure 3). Our results showed that most of the aneuploidies were trisomies identified in almost all the chromosomes, except chromosome 1, while monosomies were found only in chromosomes X and 21. Interestingly, in patients who suffered from sporadic abortion (SA), chromosome 16 was the most commonly affected, followed by chromosomes X, 22, 15, and 21. Chromosome 16 remained the most affected chromosome in patients who had undergone RMs, followed by chromosomes 22, X, 15, and 21. In the group with age < 35 years, the aneuploidy distribution was similar to that observed in the SA group. However, in the group with age ≥ 35 years, chromosome 15 was most commonly affected, followed by chromosomes 22, 16, 21, and X.

### The Correlations Among Miscarriage Frequency, Maternal Age, and Chromosomal Variants

We compared the frequency and distribution of chromosomal variants among different groups (Tables 1, 2). When maternal age was < 35 years, no significant difference was observed in the overall chromosomal variants between the SA and RM groups, with the exception of pathogenic CNVs, which are more frequent in the RM group (*P* < 0.05). When maternal age was greater than 35 years, the SA group showed higher frequency of the overall chromosomal variants. Among them, the frequency of aneuploidy increased slightly without being considered statistically significant (*P* = 0.083) (Table 1). In all SA cases, the group with advanced maternal age displayed a significantly higher frequency of overall chromosomal variants (*P* < 0.01) and aneuploidy (*P* < 0.01). In all RM cases, the group with advanced maternal age also exhibited a significantly higher frequency of aneuploidy (*P* < 0.01). However, a lower frequency of VOUS was found in the advanced maternal age group (P < 0.05).

**TABLE 1:** Distribution of chromosomal abnormalities according to the frequency of miscarriages.

Groups	Undetected variants	Detected variants							Total
		Numerical			Structural			Total	
		Aneuploidy	Polyploidy	Mosaicism	Benign CNVs	Pathogenic CNVs	VOUS		
< 35, SA	166	155	23	17	10	18	43	266	432
<35, RM	135	108	16	12	5	28	45	214	349
*P*-value	0.942	0.147	0.637	0.715	0.372	0.023*	0.196	0.942	—
≥35, SA	31	71	3	3	3	3	7	90	121
≥35, RM	40	49	2	3	3	3	6	64	104
*P*-value	0.039*	0.083	*1.000*	*1.000*	*1.000*	*1.000*	0.996	0.039*	—

The P-value in Italics is the result of Fisher’s exact test. *P < 0.05.

**TABLE 2:** Distribution of chromosomal abnormalities according to maternal age.

Groups	Undetected variants	Detected variants							Total
		Numerical			Structural			Total	
		Aneuploidy	Polyploidy	Mosaicism	Benign CNVs	Pathogenic CNVs	VOUS		
SA, <35	166	155	23	17	10	18	43	266	432
SA, ≥35	31	71	3	3	3	3	7	90	121
*P*-value	0.009*	0.000*	0.191	*0.588*	*1.000*	*0.590*	0.158	0.009*	–
RM, <35	135	108	16	12	5	28	45	214	349
RM, ≥35	40	49	2	3	3	3	6	64	104
*P*-value	0.968	0.002*	*0.388*	*1.000*	*0.392*	0.069	0.044*	0.968	–

*The P-value in Italics is the result of Fisher’s exact test.* *P < 0.05.

### Characterization of Chromosomal Mosaicisms

We analyzed 35 cases of mosaicisms with different proportions. The results showed that the proportion of mosaicism ranged from 15 to 70%. Among them, 19 cases at a proportion of 45–60% accounted for the majority of mosaicisms. Nine cases at a proportion of 60–70% accounted for the remainder of the mosaicisms, followed by three cases, respectively, at proportions of 15–30% and 30–45%. Lastly, we identified only one case with the lowest proportion of 15%.

### The Implication of Pathogenic Copy Number Variations Beyond Pregnancy Loss

**FIGURE 4:**
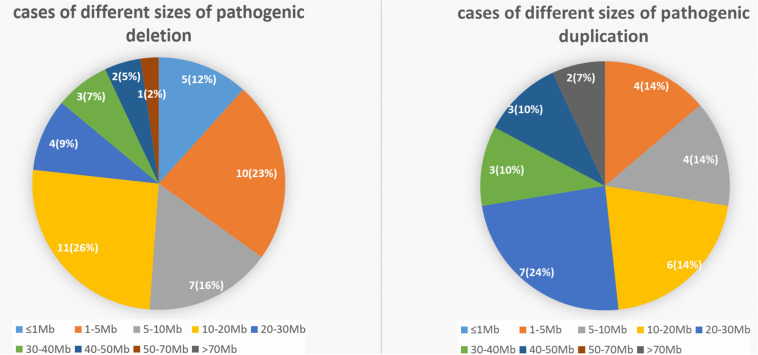
The number and percentage of cases with different sizes of pathogenic deletion/duplication.

To elucidate an association between the specific CNV and first-trimester pregnancy loss, we analyzed 52 cases with pathogenic deletion/duplication (Table 3). Among them, 15 cases had two pathogenic CNVs, one case had three pathogenic CNVs, and one case had four pathogenic CNVs concurrently. So, we identified 72 pathogenic CNVs, including 21 deletions (≥10 Mb), 21 duplications (≥10 Mb), 22 microdeletions (<10 Mb), and eight microduplications (<10 Mb). The sizes of the 72 pathogenic CNVs ranged between 0.16 and 90.56 Mb. The details of different sizes and significance are depicted in Figure 4.

**TABLE 3:** Pathogenic copy number variations identified by NGS.

Case no.	Age	Frequency of miscarriage	Del/Dup (size)(hg19)	Size	Clinical significance	Parental CNV	Parental karyotype
1	37	RM	del(16)p13.3	0.16 Mb	ATR-16 syndrome	Lost	Lost
2	34	RM	del(4)p16.3	0.26 Mb	Wolf-Hirschhorn syndrome	Not done	
3	35	RM	del(5)p13.2–p13.1	0.6 Mb	Stuve-Wiedemann syndrome (STWS)	Not done	♀:46,XX ♂:46,XY
4	28	SA	del(12)q24.33	0.68 Mb	Facial dysmorphism, Immunodeficiency, Livedo, Short stature (FILS)	Lost	Lost
5	34	SA	del(1)q21.1–q21.2	0.88 Mb	1q21.1 recurrent microdeletion syndrome	Lost	Lost
6	28	SA	del(16)p13.11	1.34 Mb	16p13.11 recurrent microdeletion syndrome	Lost	
7	25	RM	del(5)q35.3	1.61 Mb	Leukotriene c4 synthase deficiency	♀:normal ♂:normal	Lost
8	40	SA	del(7)q21.11–q21.12	1.84 Mb	Intrahepatic cholestasis of pregnancy-3 (ICP3)	♀:del(7)q21.11– q21.12(1.82 Mb) ♂:not done	♀:46,XX ♂:46,XY
9	27	SA	del(6)q15–q16.1	3.1 Mb	Short stature, Hypotonia, Microcephaly, etc.	♀:del(6)(q1 5q16.1)(3.1 Mb) ♂:normal	Lost
10	28	SA	del(2)q37.3	3.56 Mb	2q37 monosomy syndrome	Lost	Not done
11	23	RM	del(1)p36.33–p36.32	3.86 Mb	1p36 microdeletion syndrome	♀:normal ♂:normal	♀:46,XX ♂:46,XY
12	25	RM	del(4)q35.1q35.2	4.46 Mb	Patent ductus arteriosus, Ventriculomegaly, etc.	♀:normal ♂:not done	Lost
13	31	RM	del(18)p11.32–p11.31	6.6 Mb	18p deletion syndrome	Not done	Lost
14	27	RM	del(8)p23.3–p22	12.9 Mb	8p23.1 deletion syndrome	♀:normal ♂:normal	♀:46,XX ♂:46,XY
15	29	RM	del(5) p15.33–p15.2	13.14 Mb	Cri du chat syndrome (5p deletion)	Lost	Not done
16	33	RM	del(17)p13.3–p12	14.24 Mb	Miller-Dieker syndrome (MDS), 17p13.1 deletion syndrome	Not done	Not done
17	24	RM	del(14)q32.12–q32.33	14.4 Mb	Hypotonia, Genitourinary abnormalities, etc.	♀:normal ♂:normal	Not done
18	32	RM	del(1)p36.21–p36.33	14.84 Mb	1p36 deletion syndrome	Not done	♀:46,XX ♂:46,XY
19	26	SA	del(18)p11.32–p11.1	15.3 Mb	18p deletion syndrome	Lost	Not done
20	31	RM	del(1)q42.2–q44	15.78 Mb	Ventricle enlargement, hydrocephalus, callosal agenesis, etc.	Not done	
21	33	RM	del(4)p16.3–p15.1	33.12 Mb	Wolf-Hirschhorn syndrome	♀:VOUS ♂:normal	Not done
22	37	SA	del(4)p16.3–p15.1	35.42 Mb	Wolf-Hirschhorn syndrome	Not done	Not done
23	30	RM	del(8)p23.3–p11.21	40.62 Mb	8p23.1 deletion syndrome	♀:normal ♂:normal	
24	30	RM	del(8)p23.3–p11.21	41.98 Mb	8p23.1 deletion syndrome	Not done	Not done
25	28	SA	del(8) p23.3–p22 del(8) q24.22	17.54 Mb 1.24 Mb	8p23.1 deletion syndrome; Charcot-Marie-tooth disease, type 4d (CMT4D)	Not done	♀:46,XX ♂:46,XY
26	24	SA	dup(9) p24.2–p24.1	1.24 Mb	Diabetes mellitus, Neonatal, Congenital hypothyroidism (NDH)	Not done	Not done
27	28	SA	dup(22)q11.1–q11.21	1.8 Mb	Autosomal recessive, type IIC(ARCL2C)	Not done	♀: 46,XX ♂:46,XY, Y = 18
28	24	SA	dup(3) p24.2–p24.1	3.86 Mb	Congenital disorder of deglycosylation (CDDG)	Not done	
29	31	SA	dup(14) q32.2–q32.3	4.82 Mb	Mitochondrial complex IV deficiency	Lost	♀:46,XX ♂:46,XY
30	28	RM	dup(21) q22.2–q22.3	5.68 Mb	Down syndrome	♀:normal ♂:normal	
31	25	RM	dup(21)q22.11–q22.3	15.74 Mb	Down syndrome	Not done	
32	32	RM	dup(21) q21.3–q22.3	20.88 Mb	Down syndrome	♀:normal ♂:normal	
33	28	RM	dup(13) q22.2–q34	38.45 Mb	Developmental delay, Autism spectrum disorders, etc.	♀:normal ♂:not done	Not done
34	27	RM	dup(8)p23.3–p11.1	43.68 Mb	8p23.1 duplication syndrome	Not done	Not done
35	38	SA	dup(16) q11.2–q24.3	43.72 Mb	Low birth weight, Hypotonia, Epilepsy, Encephalatrophy, etc.	Lost	Not done
36	33	SA	dup(9)q13–q34.3	74.20 Mb	Growth restriction, Craniofacial deformity, etc.	Not done	Not done
37	31	RM	dup(6)p25.3–q15 dup(12)q24.31–q24.33	90.56 Mb 9.46 Mb	Intrauterine growth restriction, Microcephaly, Systemic edema, etc.; Primary autosomal recessive microcephaly-16 (MCPH16)	♀:VOUS ♂:normal	Not done
38	31	RM	del(1)p36.33–p36.32 dup(1)p36.32–p36.11	1.52 Mb 24.88 Mb	1p36 deletion syndrome; Focal facial skin dysplasia, Ectoderm lesions, etc.	♀:VOUS ♂:not done	
39	30	SA	del(14)q32.32–q32.33 dup(20) p13–p12.3	3.96 Mb 8.78 Mb	Postnatal growth retardation, Hypotonia, Severe myopia, etc.; Systemic edema, Thrombocytopenia, Anemia, etc.	Not done	
40	30	RM	del(22)q13.31–q13.3 dup(14)q31.3–q32.33	5.46 Mb 21.12 Mb	22q13 deletion syndrome; Developmental delay, Short stature, etc.	♀:normal ♂:normal	
41	28	RM	del(8) p23.3–p23.1 dup(8) p12–p11.1	6.66 Mb 12.54 Mb	8p23.1 deletion syndrome; Autosomal dominant mental retardation-32 (MRD32)	Not done	Not done
42	30	SA	del(7) p22.3–p22.1 dup(8)p23.3–p23.1	6.68 Mb 12.42 Mb	Psychomotor retardation, Ventricular septal defect, etc.; 8p23.1 duplication syndrome	♀:VOUS ♂:normal	Not done
43	35	RM	del(8) p23.3–p23.1 dup(8) p23.1–p12	6.96 Mb 22.34 Mb	8p23.1 deletion syndrome; Postnatal growth retardation, Autism, Stereotyped behavior, etc.	Not done	
44	31	RM	del(2) q37.1–q37.3 dup(1)p36.33–p36.11	9.62 Mb 24.34 Mb	2q37 monosomy syndrome; Dysgnosia, Ventricular hypertrophy, Scoliosis, etc.	Not done	Lost
45	33	RM	del(5)p15.33–p15.2 dup(16)p13.3–p11.2	10.26 Mb 32.42 Mb	Cri du chat syndrome (5p deletion); 16p13.11 recurrent microduplication, 16p11.2–p12.2 microduplication syndrome, 16p11.2 microduplication syndrome	Not done	Not done
46	28	SA	del(7)q35–q36.3 dup(18)q21.2–q23	15.94 Mb 28.4 Mb	Intellectual disability, Microcephaly, etc.; Anencephaly, Ventricular septal defect, etc.	Not done	Not done
47	26	SA	del(18)q21.32–q23 dup(15)q23–q26.3	20.4 Mb 34.46 Mb	Spinal and fibula dysplasia, Renal hypoplasia, etc.; 15q26 overgrowth syndrome	Lost	Not done
48	25	SA	del(14)q31.1–q32.33 dup(6)q25.3–q27	26.7 Mb 14.06 Mb	Psychomotor retardation, Language barrier, etc.; Developmental delay, Hypertonia, etc.	Lost	Not done
49	30	RM	del(5)p15.33–p13.3 dup(5)p13.3–p11	30.72 Mb 15.54 Mb	Cri du chat syndrome (5p deletion); 5p13 duplication syndrome	Lost	Lost
50	26	RM	del(18)q11.2–q23 dup(6)q22.31–q27	56.1 Mb 49.56 Mb	18q deletion syndrome; Microcephaly, Congenital heart disease, Renal dysplasia, etc.	♀:normal ♂:normal	Lost
51	30	SA	del(18)q22.3–q23 del(18)p11.32–p11.21 dup(18)q21.33–q22.2	6.22 Mb 14.82 Mb 8.66 Mb	Developmental delay, Epilepsy, Infantile autism, etc.; 18p deletion syndrome; Multiple Congenital Anomalies-Hypotonia- Seizures syndrome1 (MCAHS1)	♀:normal ♂:normal	Not done
52	29	RM	del(4) q32.3–q35.2 del(18) q21.2–q23 dup(16)q23.2–q24.3 dup(11)q22.3–q25	21.91 Mb 25.82 Mb 10.64 Mb 29.34 Mb	Cardiac abnormalities, Atrial septal defect, etc.; Spinal dysplasia, Kidney, and fibula dysplasia, etc.; Epilepsy, Spastic paraplegia, Spider fingers, etc.; Neurodevelopmental defects, Intellectual disability, etc.	♀:normal ♂:VOUS	Not done

*The clinical significance, referring to the databases: Berry DB, DECIPHER, OMIM, and DGV.*

In our study, the pathogenic deletions were most commonly found in the 8p23.1 region in six cases. The 18q23 region in four cases was observed to be the second most common region of pathogenic deletion. The 18p11.3, 4p16.3, 5p15, and 1p36 were the other common regions of deletion in three cases, respectively. In contrast, a high frequency of pathogenic duplication in three cases was equally observed in 21q22, 8p23.1, and 16q24.3. The clinical significance of the above CNV interpretation is shown in detail in Table 3, referring to the following databases: Berry DB, DECIPHER, OMIM, and DGV.

## Discussion

Pregnancy loss during the first trimester is triggered by many factors. The chromosomal abnormalities remain the universally acknowledged major cause (approximately 50–60%) (Goddijn and Leschot, 2000). Traditionally, G-band karyotyping has been the gold standard technique used for the detection of chromosomal aneuploidies and imbalances (Hillman et al., 2011). In the last decade, advanced molecular cytogenetic techniques, such as CMA, have been developed rapidly (Pauta et al., 2018). Notably, NGS, due to its higher resolution and high-throughput approach, has proved to be a reliable tool for chromosomal diagnosis (Martin and Warburton, 2015). Significantly, CNV analysis by NGS has been applied in several clinical studies that reported a possible association between pregnancy loss and chromosomal aberrations (Neeta Vora et al., 2016).

We used NGS to analyze CNVs in the first-trimester products of conception. Our study had a dramatically lower rate of maternal cell contamination (0.4%) when compared to an earlier study (Wang et al., 2017). The overall frequency of chromosomal variants was 63.0% (634/1,006) in our study, of which 615 (61.1%, 615/1,006) cases were considered abnormal results. Our data demonstrated that aneuploidy formed the largest proportion (60.4%) of all chromosomal aberrations detected. However, it was still lower than the frequency reported earlier (Pauta et al., 2018). Polyploidy constituted the second-largest proportion (6.9%) of numerical chromosomal variants, which was similar to the proportion reported in earlier studies (Shen et al., 2016; Wang et al., 2017). The frequency of mosaicisms was 5.5%, slightly higher than that reported previously (Sahoo et al., 2017). Additionally, submicroscopic chromosomal imbalances were reflected in 172 cases with structural variants, including 19 (3.0%) benign CNVs, 52 (8.2%) pathogenic CNVs, and 101 (16%) VOUS. Although a similar frequency of microdeletions/microduplications, as presented in this study, has been reported by earlier studies using CMA (Pauta et al., 2018), NGS has a clear advantage in being capable of detecting CNV fragments as small as 0.16 Mb. Pathogenic CNVs were represented at a lower frequency in our study than in the earlier published reports (Shen et al., 2016; Wang et al., 2017), except for one (Sahoo et al., 2017). Notably, these earlier studies highlighted that microdeletions and microduplications occurred at similar frequencies in the products of conception (Levy et al., 2014; Sahoo et al., 2017; Wang et al., 2017).

We observed a higher frequency of VOUS CNVs in our study than the earlier published studies that reported lower frequencies at 2.8, 2.0, and 2.78%, respectively (Levy et al., 2014; Pauta et al., 2018; Wang et al., 2017). Referring to the database resources, we did not find clear scientific literature to correlate the VOUS with embryonic development or death. Hence, large-scale studies are warranted to characterize the role of VOUS in the future.

We successfully identified 52 cases with 72 pathogenic CNVs. Several chromosomal regions, such as del8p23.1, del18q23, del18p11.3, del4p16.3, del5p15, del1p36, dup21q22, dup8p23.1, and dup16q24.3 presented with high-frequency deletion/duplication. Some of the regions identified in the present study have been reported earlier to be associated with pregnancy loss. Particularly, some studies have implicated high-frequency deletions in 8p23, 18p11, and 1p36 regions in cases of pregnancy loss (Shen et al., 2016; Wang et al., 2017). Furthermore, regions with high-frequency deletion reported in earlier studies, such as 6q25, 7q36.3, and 7p22.3, were observed in our study as well. Due to the consistent regions that are being reported in products of conception, future studies may reveal the genetic causes of pregnancy loss in a deeper level. It is hypothesized that the pathogenic CNVs reported through these studies may lead to crucial developmental deformities that render the fetus unviable.

Several guidelines have emphasized the necessity of fetal chromosomal examination in cases of RM (Laurino et al., 2005; Practice Committee of the American Society for Reproductive Medicine, 2012). A higher frequency of cytogenetic abnormalities has been reported in the RM group as compared with the SA group (Choi et al., 2014). In contrast, another study on 832 abortive specimens from Chinese women found no statistically significant differences in the prevalence of aneuploidy between cases of RM and SA (Jia et al., 2015). In our study, we find no significant difference in the frequency of chromosomal variants between the SA and RM groups when the maternal age is lower than 35 years. However, the RM group exhibited a higher frequency of pathogenic CNVs (28/349 vs. 18/432, *P* < 0.05). When the maternal age is more than 35 years, the SA group showed a higher frequency of overall chromosomal variants (90/121 vs. 64/104, *P* < 0.05). The inconsistent inference from multiple studies might be due to the application of different techniques, the gestational age, and the number of samples. Warburton et al. (2004) noted that the women who had previously undergone a pregnancy loss with an aneuploid abortus were at an increased risk of recurrent aneuploidies in subsequent pregnancies. Consequently, we recommend that fetal chromosome analysis should be offered routinely for all pregnancy losses, regardless of its frequency.

Maternal age is an independent risk factor for both SA and RM (Nybo Andersen et al., 2000). Two previous studies reported that women with advanced age (≥35 years) demonstrated a higher rate of chromosomal abnormalities in their fetuses (Choi et al., 2014; Wang et al., 2017). Undoubtedly, the sharp increase in the rate of pregnancy loss is partly due to the increasing rates of aneuploidies seen in older oocytes. These aneuploidies are mainly derived from non-disjunction errors during the first meiotic division of oocytes and are significantly associated with advanced maternal age (Nybo Andersen et al., 2000; Tur-Torres et al., 2017). Similarly, in our study, in the SA group, cases with advanced maternal age showed a significantly higher frequency of overall chromosomal variants (90/121 vs. 266/432, *P* < 0.01). Also, the frequency of aneuploidy was sharply increased in the advanced maternal age group (71/121 vs. 155/432, *P* < 0.01). Additionally, in the RM group, cases with advanced maternal age also showed a significantly higher frequency of aneuploidy (49/104 vs. 108/349, *P* < 0.01). In conclusion, we confirmed that the frequency of chromosomal variants, especially aneuploidy, would be increased when maternal age is greater than 35 years. It is worth noting that these aneuploidies are the most likely cause of pregnancy loss.

While investigating the source of fetal chromosomal abnormalities among the 40 cases with pathogenic deletion/duplication, we identified two microdeletions inherited from mothers, which were unlikely to cause pregnancy loss. According to our follow-up, the two mothers have not shown an abnormal phenotype for the time being. The etiology of pregnancy loss is complex and may be related to unrecognized uterine pathology, immune factors, infection, inflammation, underlying pathological conditions, etc. It appears more likely that fetal chromosomal abnormalities occur *de novo* rather than being inherited (Carp et al., 2006; van den Berg et al., 2012).

## Data Availability Statement

The datasets presented in this study can be found in online repositories. The names of the repository/repositories and accession number(s) can be found below: https://www.ebi.ac.uk/dgva/, 1006.

## Ethics Statement

The studies involving human participants were reviewed and approved by Ethics Committee of Tongji Hospital, Tongji Medical College, Huazhong University of Science and Technology. The patients/participants provided their written informed consent to participate in this study.

## Author Contributions

JX contributed to conception and supervision. SC, WZ, and DD contributed to data collection. SL, XX, and XS contributed to data analyses. YW, SC, and LFe contributed to checking the analyses. JW and JX contributed to manuscript revision. LFa contributed to manuscript writing. All authors contributed to the article and approved the submitted version.

## Conflict of Interest

The authors declare that the research was conducted in the absence of any commercial or financial relationships that could be construed as a potential conflict of interest.
